# An updated meta-analysis investigating the association between DNMTs gene polymorphism andgastric cancer risk

**DOI:** 10.1371/journal.pone.0293466

**Published:** 2023-10-25

**Authors:** Yu-Long Zhang, Yu-Wei Wang, Ming-Jie He, Jian-Lan Chang

**Affiliations:** 1 Department of Oncology, Heping Hospital Affiliated to Changzhi Medical College, Shanxi, Changzhi, China; 2 Changzhi Medical College, Shanxi Province, Changzhi, China; Indiana University Purdue University at Indianapolis, UNITED STATES

## Abstract

Gastric cancer (GC) is a prominent global health issue, as it ranks as the fifth most prevalent type of cancer and the fourth most significant cause of cancer-related mortality worldwide. Although H. pylori is known to play a role in the development of GC, genetic factors also play a role in its onset and progression. Recent studies have shown that genetic polymorphisms are strongly associated with the development of GC and that certain single nucleotide polymorphisms (SNPs) can be used as biomarkers for early diagnosis and prevention. Epigenetic disturbances, such as DNA methylation, are involved in the development of GC, and mutations in the DNA methyltransferase (DNMT) gene have been found to increase the risk of GC. However, previous findings on the association between DNMTs SNPs and GC risk have been inconsistent. In this study, an updated meta-analysis of three well-studied and controversial DNMTs polymorphic loci, DNMT1 rs16999593, DNMT3A rs1550117 and DNMT3B rs1569686, was performed to provide more reliable results. It was found that DNMT1 rs16999593 was not associated with GC, DNMT3A rs1550117 may have a positive association with GC risk, and DNMT3B rs1569686 may be a protective factor for GC. These findings may provide valuable information for early diagnosis and prevention of GC, but further studies are needed to confirm these results.

## Introduction

Gastric cancer (GC) is a major health problem worldwide, with an annual incidence of over one million and causing more than 700,000 deaths globally [1, 2]. Although the specific mechanisms underlying the development of GC are still not well understood, epidemiological studies and a vast amount of past experimentation suggest that Helicobacter pylori may play a key role in the pathogenesis of GC. However, not all H. pylori infections necessarily lead to GC, as only about 1–3% of infected individuals ultimately develop the disease, indicating that genetic factors also play an important role in the onset and progression of GC [3]. Current research suggests that genetic polymorphisms are closely associated with the occurrence and development of GC, and certain single nucleotide polymorphisms (SNPs) may serve as important biological markers for the early diagnosis and prevention of GC.

The development of GC is the result of the interaction between genetics and epigenetics [4]. In epigenetics, DNA methylation is an important regulatory mechanism that is involved in gene transcription regulation and chromatin remodeling [5]. Recent studies have found that epigenetic interference, due to the functional impairment of DNMTs genes, is involved in tumorigenesis and progression [6]. In mammals, five members of the DNMT protein family are known, including DNMT1, DNMT2, DNMT3A, DNMT3B, and DNMT3L, among which only DNMT1, DNMT3A, and DNMT3B have been found to have DNA methyltransferase activity [7]. DNMT1 is usually referred to as a maintenance methyltransferase and is responsible for maintaining the pre-existing methylation pattern during DNA replication [8, 9]. DNMT3A and DNMT3B are considered de novo DNA methyltransferases, and play a crucial role in the occurrence of GC [10, 11]. In addition, abnormal promoter methylation is also involved in the development of human GC in various tumor suppressor genes [12].

Recent studies suggest that mutations in DNMTs genes are associated with an increased risk of GC. However, previous research results are inconsistent and even contradictory. Some studies, such as those conducted by Yang et al. [13], Gao et al. [14], and Li et al. [15], have found that the rs16999593 polymorphism in DNMT1 is associated with susceptibility to GC, but Jiang et al. [16] and Zhou et al [17]. believe that there is no correlation between the two. In addition, some meta-analyses have not yet reached a conclusion on the effect of DNMTs SNPs on GC, and there have been new publications on the relationship between DNMTs and GC risk. Therefore, this study selected three DNMTs polymorphic loci with more research and controversy, DNMT1 rs16999593, DNMT3A rs1550117, and DNMT3B rs1569686, for an updated meta-analysis to provide more reliable results on these issues.

## Materials and methods

### Search strategy

Based on the PRISMA guidelines [18], we conducted a meta-analysis on the relationship between DNMT polymorphisms and GC risk. We searched several databases, including PubMed, EMBASE, and the China National Knowledge Infrastructure, using the following keyword strategy: (stomach neoplasms OR gastric neoplasms OR stomach tumors OR gastric tumors OR stomach cancers OR gastric cancers OR stomach carcinomas OR gastric carcinomas) AND (DNMT1 OR DNMT3A OR DNMT3B OR DNMTs OR DNA methyltransferases) AND (polymorphism OR variant OR mutation OR genotype OR allele). Our search was current up until January 2023.

### Selection criteria

Inclusion criteria were as follows: (1) case-control or cohort studies; (2) studies that described the correlation between rs16999593, rs1550117, and rs1569686 polymorphisms and GC risk; and (3) studies that provided sufficient genotype data for both case and control groups. Exclusion criteria were: (1) duplicate studies; (2) studies without available data; and (3) case reports, reviews, letters, and meta-analyses.

### Data extraction

The data extraction table for this study has already been prepared in advance. Based on the established inclusion and exclusion criteria, the data was independently extracted and cross-checked. If there were any disagreements, they were discussed and negotiated until a consensus was reached. We will invite a third author to extract the data again and conduct a final check and confirmation. If the data is not detailed or there are any questions, we will try to contact the original authors to supplement and confirm the accuracy and completeness of the data. Each study collected the following items: country, region, study type, race, matching criteria, age, polymorphisms, number of cases and controls. Data on all polymorphisms included in the study were also extracted, including data on genotype distribution and relative risk.

### Quality assessment

The quality of all eligible studies was independently assessed by the two authors (For details of the evaluation details, please refer to the S1 Table). We designed quality assessment criteria on the basis of previous meta-analyses [15, 19–23]. S1 Table lists the scale for quality assessment of molecular association studies of GC risk. The total score was 20 points, studies scoring above 12 were excellent, those scoring less than 9 were poor and those scoring between 9 and 12 were moderate.

### Statistical analysis

This text aims to evaluate the association between DNMTs and GC. For each included study, the strength of the association was assessed by calculating the corresponding odds ratio (OR) and 95% confidence interval (CI). A significance level of P < 0.05 was considered to be statistically significant. To comprehensively evaluate different genetic models, five genetic models were compared, including: (1) allele model; (2) additive model; (3) dominant model; (4) recessive model; and (5) over-dominant model. The Hardy-Weinberg equilibrium (HWE) was examined using the chi-square goodness-of-fit test. If P > 0.05, the control group was considered to be in HWE. The heterogeneity test was conducted using the Chi-square-based Q-test and *I*^*2*^ test. When P > 0.10 and/or *I*^*2*^≤ 50%, there was no significant heterogeneity between the studies, and a fixed-effects model was used [24]. Otherwise, a random-effects model would be selected [25]. Sensitivity analysis was conducted to evaluate the stability of the results, which was estimated using the following three methods: (1) deleting one single study each time; (2) excluding low-quality and HWD (Hardy Weinberg Disequilibrium) studies; (3) selecting studies that meet the following conditions: high-quality, HWE (Hardy Weinberg Equilibrium), and matched studies. Begg’s funnel plot [26] and Egger’s test were used to assess publication bias [27]. When there was significant publication bias, the nonparametric "trim-and-fill" method was used to correct and identify the asymmetry of the funnel plot caused by publication bias, while estimating the true value of quantitative synthesis [28]. In addition, the false positive report probability (FPRP) test [29] and Venice criteria [30] were used to evaluate the credibility of statistically significant results. All statistical analyses were conducted using Stata 12.0 software.

## Results

### Description of included studies

**Fig 1:**
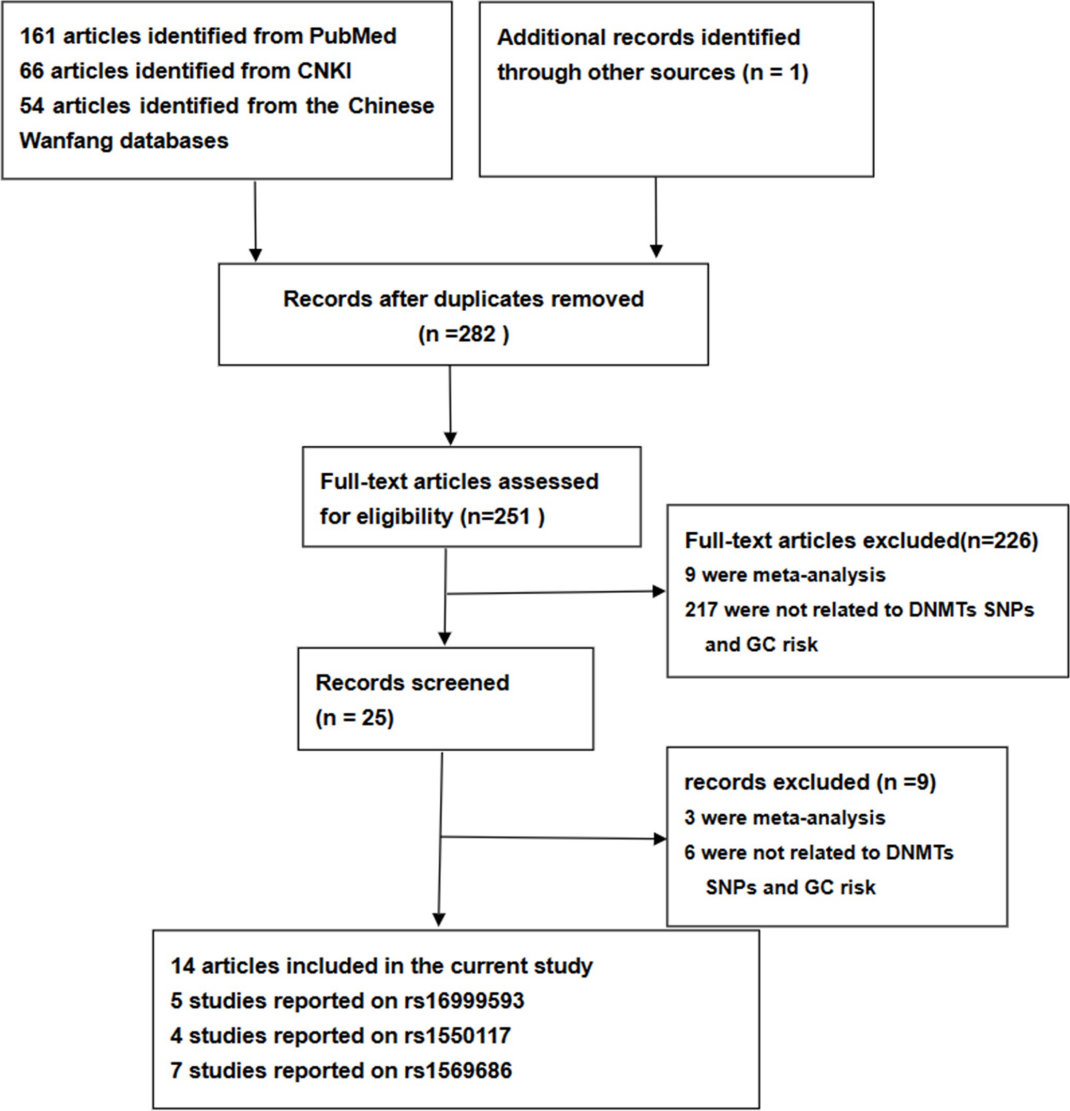
Flow diagram of the literature search.

We conducted a search based on our inclusion and exclusion criteria, resulting in 282 articles. After applying our criteria, we selected 14 studies that met our requirements, involving a total of 3539 cases of GC and 6106 controls. Within these studies, 5 investigated the association between rs16999593 and GC risk, involving 1846 cases of GC and 2554 controls; 4 studies reported on rs1550117, involving 1363 cases of GC and 2134 controls; and 7 studies investigated rs1569686, involving 1932 cases of GC and 4149 controls. Regarding quality, there were 2 high-quality studies and 3 moderate-quality studies regarding rs16999593, 2 high-quality studies and 2 moderate-quality studies regarding rs1550117, and 2 high-quality studies, 4 moderate-quality studies, and 1 moderate-quality study regarding rs1569686. We present the detailed characteristics and scores of each study in Table 1, and the selection and inclusion process of the literature in Fig 1. Furthermore, Tables 2–4 displays the genotype frequencies and HWE test results of rs16999593, rs1550117, and rs1569686 in relation to GC risk.

**Table 1:** Characteristics of 14 studies included in the meta-analysis.

Author/Year	First Province/Country	Area	Source of controls	Type of control	Gene SNPs	Sample size	HWE (controls)	Score
Zhang et al. 2008 [31]	Jiangsu/(China)	South China	HB	Non-gastric cancer Controls	DNMT3B rs1569686	156/156	0.010	9
Fan et al. 2010 [32]	Jiangsu/(China)	South China	HB	Healthy controls	DNMT3A rs1550117	208/364	0.206	13
Hu et al. 2010 [33]	Jiangsu/(China)	South China	HB	Healthy controls	DNMT3B rs1569686	259/262	0.901	13
Yang et al., 2012 [13]	Jiangxi(China)	South China	HB	Non-gastric cancer Controls	DNMT1 rs16999593	242/294	0.120	12
					DNMT3A rs1550117	242/294	0.747	
Jiang et al. 2012 [34]	Jilin(China)	North China	PB	Healthy controls	DNMT1 rs16999593	447/961	0.758	14
Cao et al. 2013 [35]	Jilin(China)	North China	PB	Healthy controls	DNMT3A rs1550117	447/961	0.932	14
Zhang et al. 2014 [36]	Heilongjiang(China)	North China	HB	Healthy controls	DNMT3B rs1569686	50/60	0.389	6
Wang et al. 2015 [37]	Jilin(China)	North China	PB	Healthy controls	DNMT3B rs1569686	447/961	0.321	9
Gao et al. 2015 [14]	Shandong(China)	North China	PB	Healthy controls	DNMT1 rs16999593	310/420	0.039	9
Chen et al. 2017 [20]	Hubei(China)	South China	PB	Non-gastric cancer Controls	DNMT3B rs1569686	460/800	0.693	14
Ahmadi et al.2018 [38]	Lorestan (Iran)	Iran	HB	Healthy controls	DNMT3B rs1569686	100/112	0.062	12
Zhou et al. 2018 [17]	Jiangsu/(China)	South China	HB	Healthy controls	DNMT1 rs16999593	466/452	0.398	12
					DNMT3A rs1550117	466/452	0.879	
Liu et al. 2018 [22]	Inner Mongolia(China)	North China	PB	Healthy controls	DNMT1 rs16999593	381/427	0.957	13
Wang et al. 2019 [21]	Hubei(China)	South China	HB	Healthy controls	DNMT3B rs1569686	460/1798	0.693	10

HB, hospital-based studies PB, population-based studies; DNMTgenes, deoxyribonucleic acidmeth-yltransferase genes; SNPs, single nucleotide polymorphisms;HWE, Hardy-Weinberg equilibrium.

**Table 2:** Meta-analysis of the association of *DNMT1 (rs16999593)* polymorphism with risk of gastric cancer.

Variable	n (Cases/Controls)		CCvs.TT^a^		TC+CCvs.TT^b^		CCvs.TC+TT^c^		TCvs.TT^d^		Cvs.T^e^	
			OR (95% CI)	Ph/I^2^ (%)	OR (95% CI)	Ph/I^2^ (%)	OR (95% CI)	Ph/I^2^ (%)	OR (95% CI)	Ph/I^2^ (%)	OR (95% CI)	Ph/I^2^ (%)
**Overall**	**5 (1846/2554)**	**REM**			**1.148(0.911–1.446)**	**0.013/68.5**			**1.149 (0.894–1.477)**	**0.008/70.9**	**1.116(0.940–1.324)**	**0.045/59.0**
		**FEM**	**1.179 (0.872–1.594)**	**0.690/0.00**			**1.117(0.829–1.505)**	**0.730/0.00**				
**North/south(china)**												
**North**	**3(1138/1808)**	**REM**			**1.120(0.774–1.621)**	**0.005/80.9**			**1.115(0.945–1.316)**	**0.004/82.3**	**1.116 (0.851–1.463)**	**0.020/74.3**
		**FEM**	**1.351 (0.927–1.968)**	**0.720/0.00**			**1.282 (0.883–1.862)**	**0.778/0.00**				
**South**	**2(708/746)**	**REM**			**1.185(0.851–1.651)**	**0.133/55.7**			**1.223(0.867–1.725)**	**0.136/55.0**		
		**FEM**	**0.938 (0.568–1.550)**	**0.586/0.00**			**0.881 (0.536–1.447)**	**0.752/0.00**			**1.090 (0.910–1.306)**	**0.175/45.7**
**Source of control**												
**PB**		**REM**			**1.120(0.774–1.621)**	**0.005/80.9**			**1.115(0.945–1.316)**	**0.004/82.3**	**1.116 (0.851–1.463)**	**0.020/74.3**
		**FEM**	**1.351 (0.927–1.968)**	**0.720/0.00**			**1.282 (0.883–1.862)**	**0.778/0.00**				
**Type of control**												
**Healthy**	**4(1584/2260)**	**REM**			**1.095(0.841–1.427)**	**0.012/72.6**			**1.078 (0.814–1.426)**	**0.015/71.4**	**1.804 (0.886–1.326)**	**0.031/66.1**
		**FEM**	**1.190 (0.859–1.650)**	**0.528/00.0**			**1.145(0.829–1.581)**	**0.599/0.00**				
**Egger’s test**												
***P***_***E***_			**0.433**		**0.958**		**0.468**		**0.892**		**0.822**	

PB = population-based studies;REM = Random effects model,FEM = Fixed effects model. ^a^ additive model; ^b^ dominant model; ^c^ recessive model; ^d^ over-dominant model;^e^ allele model.

**Table 3:** Meta-analysis of the association of *DNMT3A(rs1550117)* polymorphism with risk of gastric cancer.

Variable	n (Cases/Controls)		AA vs.GG^a^		AA+AG vs.GG^b^		AAvs.AG+GG^c^		AGvs.GG^d^		Avs.G^e^	
			OR (95% CI)	Ph/I^2^ (%)	OR (95% CI)	Ph/I^2^ (%)	OR (95% CI)	Ph/I^2^ (%)	OR (95% CI)	Ph/I^2^ (%)	OR (95% CI)	Ph/I^2^ (%)
**Overall**	**4 (1363/2134)**	**REM**	**1.700 (0.696–4.419)**	**0.000/83.6**	**1.155(0.905–1.474)**	**0.044/62.9**	***3*.*928(2*.*116–7*.*295)***	**0.034/65.5**			**1.201(0.889–1.623)**	**0.001/82.9**
		**FEM**							**1.074(0.924–1.249)**	**0.520/00.0**		
**North/South(china)**												
**south**	**3 (961/1108)**	**REM**	***1*.*243(1*.*004–1*.*538)***	**0.000/87.3**	**1.190(0.820–1.726)**	**0.019/74.8**	***4*.*451(1*.*891–10*.*478)***	**0.025/73.0**			**1.255 (0.804–1.958)**	**0.000/87.9**
		**FEM**							**1.064(0.878–1.288)**	**0.327/10.5**		
**North**	**1 (447/961)**		**1.047(0.582–1.982)**		**1.090(0.861–1.380)**		***2*.*848(1*.*531–5*.*296)***		**1.092(0.855–1.394)**		**1.070 (0.875–1.310)**	
**Source of control**												
**HB**	**3 (961/1108)**	**REM**	***1*.*243(1*.*004–1*.*538)***	**0.000/87.3**	**1.190(0.820–1.726)**	**0.019/74.8**	***4*.*451(1*.*891–10*.*478)***	**0.025/73.0**	**1.067(0.870–1.308)**	**0.327/10.5**	**1.255 (0.804–1.958)**	**0.000/87.9**
		**FEM**										
**Type of control**												
**Healthy**	**3(1121/1777)**	**REM**	**1.836(0.564–5.980)**	**0.000/89.9**	**1.208(0.881–1.657)**	**0.023/73.6**	***4*.*026(1*.*770–9*.*156)***	**0.013/77.0**	**1.097(0.930–1.294)**	**0.387/0.00**	**1.258(0.849–1.864)**	**0.000/88.2**
		**FEM**										
**Egger’s test**												
***P***_***E***_			**0.647**		**0.537**		**0.629**		**0.718**		**0.565**	

HB = hospital-based studies;REM = Random effects model,FEM = Fixed effects model. ^a^ additive model; ^b^ dominant model; ^c^ recessive model; ^d^ over-dominant model;^e^ allele model.

**Table 4:** Meta-analysis of the association of *DNMT3B(rs1569686)* polymorphism with risk of gastric cancer.

Variable	n (Cases/Controls)		GG vs.TT^a^		GG+GT vs.TT^b^		GGvs.GT+TT^c^		GTvs.TT^d^		Gvs.T^e^	
			OR (95% CI)	Ph/I^2^ (%)	OR (95% CI)	Ph/I^2^ (%)	OR (95% CI)	Ph/I^2^ (%)	OR (95% CI)	Ph/I^2^ (%)	OR(95%CI)	Ph/I^2^ (%)
**Overall**	**7(1932/4149)**	**REM**			***0*.*644(0*.*475–0*.*871)***	**0.002/71.8**			***0*.*641(0*.*471–0*.*871)***	**0.003/70.3**	***0*.*694(0*.*528–0*.*912)***	**0.001/73.6**
		**FEM**	***0*.*619(0*.*406–0*.*944)***	**0.644/0.00**			**0.797(0.550–1.154)**	**0.371/7.10**				
**Iran**	**1(110/112)**		**0.769(0.384–1.538)**		**0.629(0.332–1.190)**		**1.174(0.681–2.024)**		**0.482(0.231–1.006)**		**0.912(0.618–1.345)**	
**North**	**2(497/1021)**		**1.112(0.378–3.278)**		**1.085(0.683–1.723)**		**1.076(0.366–3.166)**		**1.085(0.675–1.742)**		**1.098(0.758–1.590)**	
**South**	**4 (1335/3016)**	**REM**										
		**FEM**	***0*.*433(0*.*235–0*.*795)***	**0.907/0.00**	***0*.*568(0*.*476–0*.*677)***	**0.579/0.00**	***0*.*481(0*.*262–0*.*883)***	**0.904/0.00**	***0*.*580(0*.*484–0*.*695)***	**0.627/0.00**	***0*.*593(0*.*505–0*.*696)***	**0.481/0.00**
**HB**	**5(1025/2388)**	**REM**										
		**FEM**	***0*.*564(0*.*340–0*.*934)***	**0.586/0.00**	***0*.*557(0*.*452–0*.*685)***	**0.755/0.00**	**0.824(0.536–1.267)**	**0.217/32.5**	***0*.*555(0*.*447–0*.*690)***	**0.788/0.00**	***0*.*629(0*.*526–0*.*715)***	**0.163/38.7**
**Type of control**												
**Healthy**	**5(1316/3193)**	**REM**			***0*.*745(0*.*626–0*.*888)***	**0.001/77.8**			***0*.*658(0*.*421–1*.*027)***	**0.001/78.0**	**0.757(0.529–1.083)**	**0.002/77.1**
		**FEM**	**0.701(0.433–1.135)**	**0.668/00.0**			**0.941(0.620–1.429)**	**0.497/0.00**				
**Egger’s test**												
***P***_***E***_			**0.212**		**0.459**		***0*.*044***		**0.386**		**0.524**	

HB = hospital-based studies, REM = Random effects model,FEM = Fixed effects model. ^a^ additive model; ^b^ dominant model; ^c^ recessive model; ^d^ over-dominant model;^e^ allele model.

### Meta-analysis results

The association results between DNMT1 rs16999593 and cancer risk are shown in Table 2. Overall, no correlation was found between the genetic models and GC risk (CC vs. TT: OR 1.179, 95% CI 0.872–1.594; TC+CC vs. TT: OR 1.148, 95% CI 0.911–1.446; CC vs. TC+TT: OR 1.170, 95% CI 0.829–1.505; TC vs. TT: OR 1.149, 95% CI 0.940–1.324).

Table 3 summarizes the evaluation results of the association between DNMT3A rs1550117 polymorphism and GC risk. We observed an increased risk of GC (AA vs. AG+GG: OR 3.928, 95% CI 2.116–7.295). Through subgroup analysis by ethnicity, we observed an increased risk of GC in the additive model (AA vs. GG: OR 1.243, 95% CI 1.004–1.538) in the Chinese southern population, in addition to the recessive model.

Overall analysis showed a significant reduction in GC risk in the rs1569686 genotypes (GG vs. TT: OR 0.619, 95% CI 0.406–0.994; GG+GT vs. TT: OR 0.568, 95% CI 0.476–0.677; GT vs. TT: OR 0.641, 95% CI 0.471–0.871; G vs. T: OR 0.694, 95% CI 0.262–0.883) (Table 4). Using ethnic subgroup analyses, we observed that all genetic models of DNMT3B rs1550117 polymorphism reduced the risk of GC in the southern Chinese population, but not in the northern Chinese population.

### Heterogeneity and sensitivity analyses

**Fig 2:**
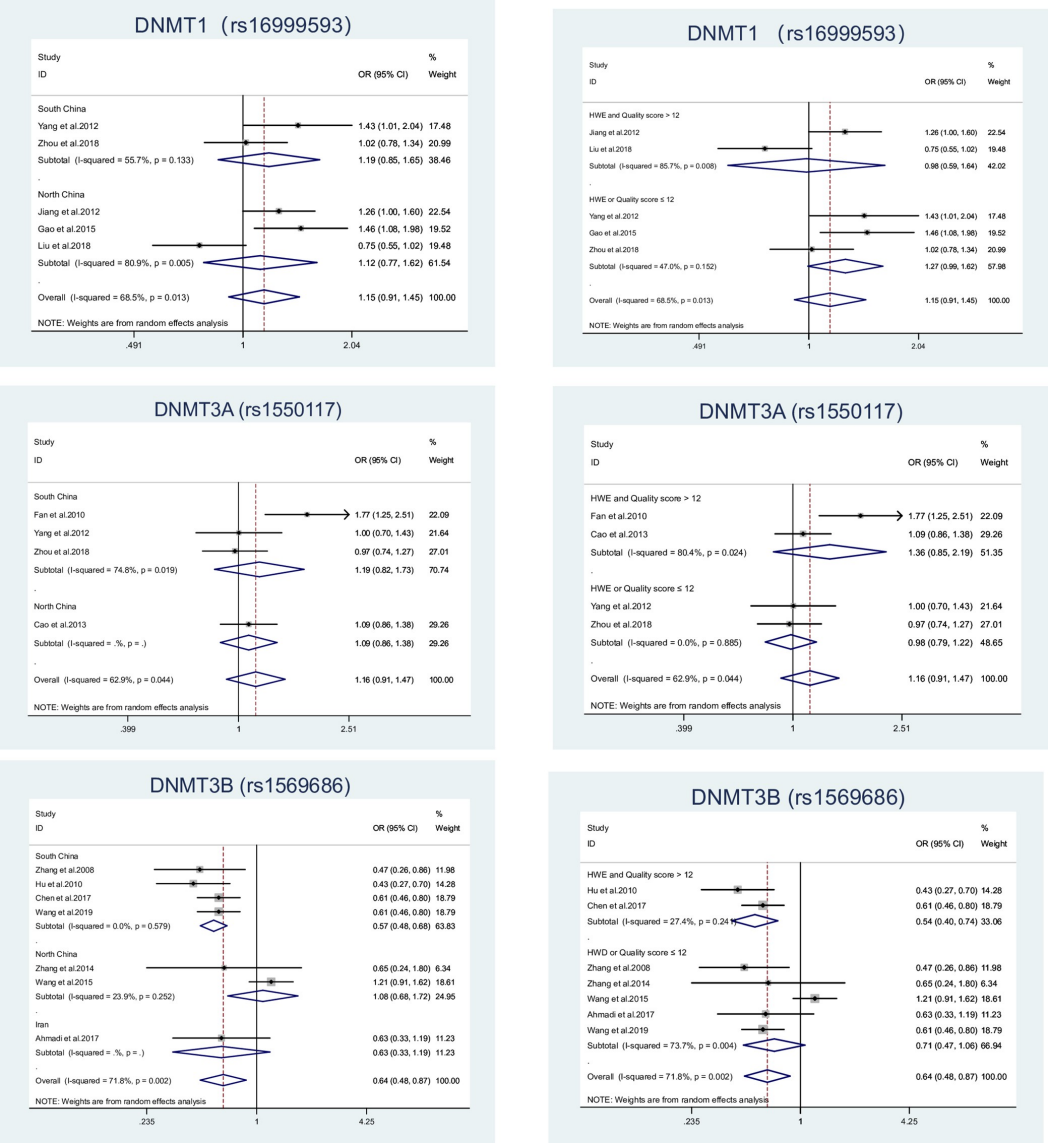
Forest plot of subgroup analysis on DNMT1 rs16999593、DNMT3A rs1550117 and DNMT3B rs1569686 polymorphisms (dominant model) by population area and study quality. Population area(South China,North China and Iran) (A); Study quality (HWE and Quality score>12 and HWE and Quality score≤12) (B).

Heterogeneity was observed in all three SNPs, both overall and in multiple subgroup analyses. Among them, rs16999593 exhibited noteworthy heterogeneity in three genetic models: TC+CC vs. TT (*I*^*2*^ 68.5%, Phet 0.013), TC vs. TT (*I*^*2*^ 70.9%, Phet 0.008) and C vs. T (*I*^*2*^ 59.0%, Phet 0.045). Similarly, heterogeneity was evident in rs1550117, including AA vs. GG (*I*^*2*^ 83.6%, Phet 0.000), AA+AG vs. GG (*I*^*2*^ 62.9%, Phet 0.044), AA vs. AG+GG (*I*^*2*^ 65.5%, Phet 0.034) and A vs. G (*I*^*2*^ 82.9%, Phet 0.001). Furthermore, rs1569686 was also found to be heterogeneous, including GG+GT vs. TT (*I*^*2*^ 71.8%, Phet 0.002), GT vs. TT (*I*^*2*^ 70.3%, Phet 0.003) and G vs. T.The sensitivity analysis demonstrated that the study conducted by Liu et al [9] was the primary influence for rs16999593 heterogeneity, whereby 95% CI became positive in the direction and heterogeneity became significantly lower(*I*^*2*^ 20.6%, Phet 0.287). For rs1550117, the study conducted by Fan et al [32] was also the study that mainly caused heterogeneity because when that study was excluded, AA vs. AG+GG: *I*^*2*^ 0.00%, Phet 0.780. Similarly, the study conducted by Wang et al [37] was the study that mainly caused heterogeneity after the exclusion of rs1569686, whereby 95% CI did not change in the direction change, but zero heterogeneity was observed: *I*^*2*^ 0.00%, Phet 0.832.We compared the characteristics of the three studies and conducted subgroup analyses using population area and study quality as two factors explaining heterogeneity (Fig 2). In the South China region, rs1569686 was negatively associated with GC risk (GG+GT vs. TT: OR 0.57, 95% CI 0.48–0.68); however, in the North China region and Iran, rs1569686 was not associated with GC risk (GG+GT vs. TT: OR 1.08, 95% CI 0.68–1.72; OR 0.63. 95% CI 0.33–1.19).

### Publication bias

**Fig 3:**
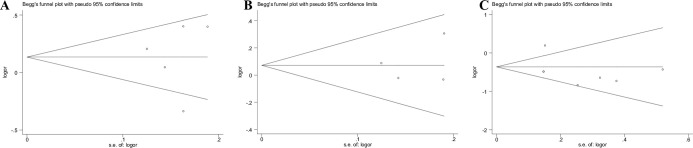
Begg’s funnel plot to assess publication bias on the combined effects of DNMTs polymorphisms with GC risk in overall population(dominant model). **(A)**:DNMT1 (rs16999593);**(B)**:DNMT3A(rs1550117);**(C)**:DNMT3B(rs1569686).

Therefore, we assessed publication bias in the literature of this study, using Begg’s funnel plot and Egger’s test. Despite the small size of the study, funnel plots can still be used to suspect the presence of publication bias by looking at the shape of asymmetries or the lack of small studies. In the present study, the shape of the funnel plot showed no significant asymmetry in the overall population (Fig 3). The results of Egger’s test showed that only the rs1569686 polymorphism was associated with publication bias in the risk of GC (GGvs. GT+TT: P = 0.044, see Table 4). To adjust for publication bias, we used a nonparametric "trim and fill" approach.

### Credibility of the identified genetic associations

In this meta-analysis, we employed a set of criteria to classify significant associations as "positive results". These criteria included a P-value less than 0.05 in at least two genetic models, FPRP less than 0.2 at the P-value level of 0.05, statistical power > 0.8, and *I*^*2*^ < 50%. Associations were classified as "positive result with low confidence" if the P-value < 0.05 in at least one genetic model, the statistical efficacy was between 50% and 79%, or the FPRP > 0.2, or the *I*^*2*^> 50%. Otherwise, the association was categorized as a "negative result". Following the credibility assessment, we identified "positive results with low credibility" in this meta-analysis. The detailed results of the credibility assessment can be found in the Table 5.

**Table 5:** Credibility of the current meta-analysis.

Variables	Model	OR (95% CI)	I2 (%)	Statistical power	Credibility	
					Prior probability of 0.001	
					FPRP	BFDP
**(DNMT3A(rs1550117)**						
**Overall**	**AAvs.AG+GG**	**3.928(2.116–7.295)**	**65.5**	**0.001**	**0.928**	**0.622**
**South China**	**AA vs.GG**	**1.243(1.004–1.538)**	**87.3**	**0.958**	**0.979**	**0.999**
	**AAvs.AG+GG**	**4.451(1.891–10.478)**	**73.0**	**0.006**	**0.990**	**0.977**
**HB**	**AA vs.GG**	**1.243(1.004–1.538)**	**87.3**	**0.958**	**0.979**	**0.999**
	**AAvs.AG+GG**	**4.451(1.891–10.478)**	**73.0**	**0.006**	**0.990**	**0.977**
**Healthy**	**AAvs.AG+GG**	**4.026(1.770–9.156)**	**77.0**	**0.009**	**0.990**	**0.980**
**DNMT3B(rs1569686)**						
**Overall**	**GG vs.TT**	**0.619(0.406–0.944)**	**0.00**	**0.365**	**0.986**	**0.997**
	**GG+GT vs.TT**	**0.644(0.475–0.871)**	**71.8**	**0.411**	**0.912**	**0.988**
	**GTvs.TT**	**0.641(0.471–0.871)**	**70.3**	**0.401**	**0.918**	**0.989**
	**Gvs.T**	**0.694(0.528–0.912)**	**73.6**	**0.613**	**0.935**	**0.994**
**South China**	**GG vs.TT**	**0.433(0.235–0.795)**	**0.00**	**0.082**	**0.988**	**0.992**
	**GG+GT vs.TT**	**0.568(0.476–0.677)**	**0.00**	**0.037**	**0.000**	**0.000**
	**GGvs.GT+TT**	**0.481(0.262–0.883)**	**0.00**	**0.146**	**0.992**	**0.825**
	**GTvs.TT**	**0.580(0.484–0.695)**	**0.00**	**0.066**	**0.000**	**0.000**
	**Gvs.T**	**0.593(0.505–0.696)**	**0.00**	**0.076**	**0.000**	**0.000**
**HB**	**GG vs.TT**	**0.564(0.340–0.934)**	**0.00**	**0.258**	**0.900**	**0.997**
	**GG+GT vs.TT**	**0.557(0.452–0.685)**	**0.00**	**0.045**	**0.001**	**0.002**
	**GTvs.TT**	**0.555(0.447–0.690)**	**0.00**	**0.049**	**0.002**	**0.003**
	**Gvs.T**	**0.629(0.526–0.715)**	**38.7**	**0.187**	**0.000**	**0.000**
**Healthy**	**G+GT vs.TT**	**0.745(0.626–0.888)**	**77.8**	**0.893**	**0.532**	**0.970**
	**GTvs.TT**	**0.742(0.619–0.889)**	**78.0**	**0.877**	**0.580**	**0.974**
**HWE and Quality score > 12**	**GG+GT vs.TT**	**0.565(0.451–0.709)**	**0.00**	**0.077**	**0.011**	**0.041**
**Overall**	**GTvs.TT**	**0.555(0.438–0.703)**	**0.00**	**0.064**	**0.016**	**0.051**
	**Gvs.T**	**0.653(0.470–0.908)**	**60.6**	**0.451**	**0.962**	**0.995**

HB = hospital-based studies,BFDP = Bayesian False Discovery Probability,FPRP = false positive report probability.

## Discussion

Epigenetic modifications constitute a pivotal natural process during normal developmental stages. However, it is noteworthy that aberrant epigenetic modifications may engender harmful effects, and ultimately lead to the onset and progression of cancer [39]. Epigenetic alterations, including histone modifications, non-coding RNA, and DNA methylation, are widely acknowledged to trigger the inactivation of oncogenes and other genes that are associated with GC [40–42]. There is mounting evidence indicating that genetic variations in DNMTs, particularly SNPs, and their haplotype blockade, are associated with the incidence of numerous cancers, including GC. SNPs can alter the activity of promoters, the regulation of gene expression, splice sites, transcription factor binding sites, and epigenetic modifications [43]. Hence, the identification of related polymorphisms may serve as potential biomarkers for predicting GC. Despite numerous studies exploring the relationship between genetic polymorphisms in DNMTs and GC risk, no conclusive evidence has been obtained. This can be attributed to factors such as small sample sizes, ethnic and regional differences, among others. To overcome these limitations, meta-analysis represents an effective alternative approach.

Six previous meta-analysis studies [15, 19–23] have explored the association between genetic polymorphisms in DNMTs and GC risk (S2 Table). rs16999593 was found to be associated with an increased risk of GC by Li et al [15], Neves et al [19] and Li et al [23]. In addition, Li et al [15] analyzed three rs1550117 studies and five rs1569686 studies and showed that the rs1550117 polymorphism was associated with an increased risk of GC, while the rs1569686 polymorphism was associated with a decreased risk of GC. However, a study by Wang et al [21] found that the rs1550117 polymorphism was not associated with an increased risk of GC. We carefully examined the shortcomings of these meta-analyses. First, quality assessment of eligible studies was not performed in all previous studies[15, 19–23] which may have led to the inclusion of low-quality literature in these meta-analyses, thus biasing the results. Second, for genetic association studies, the HWE test is necessary to ensure that the distribution of genotypes in the control group is as expected. If the controls do not conform to HWE, selection bias or genotyping errors may occur, leading to misleading results. Some previous meta-analysis studies did not have control group genotype distribution for HWE testing [14]. Then, some previous meta-analysis studies did not calculate statistical power. Finally, all previous meta-analyses also did not assess the probability of statistically significant association of false positive reports. Therefore, the results of these meta-analyses may not be credible. In addition, we assessed the credibility of the identified genetic associations in these previous meta-analyses (Table 6) and identified statistically significant correlations with "less credible positive results" in the previous meta-analyses.

**Table 6:** Credibility of the current meta-analysis.

Study	SNPs	Model	OR (95% CI)	I2 (%)	Statistical power	Credibility	
						Prior probability of 0.001	
						FPRP	BFDP
**Li et al. 2016**	***DNMT1 rs16999593***	**TCvs. TT**	**1.36 (1.14,1.61)**	**0.00**	**0.872**	**0.289**	**0.999**
		**TC/CC vs. TT**	**1.36 (1.15,1.60)**	**0.00**	**0.881**	**0.191**	**0.891**
	***DNMT3A rs1550117***	**AA vs. GG**	**2.03 (1.38,3.00)**	**86.9**	**0.064**	**0.855**	**0.917**
		**GA/AA vs. GG**	**1.20 (1.01,1.42)**	**69.0**	**0.995**	**0.971**	**0.999**
		**AA vs. GA/GG**	**1.96 (1.33,2.89)**	**85.8**	**0.088**	**0.885**	**0.947**
	***DNMT3B rs1569686***	**GT/GG vs. TT**	**0.74 (0.61,0.90)**	**80.1**	**0.852**	**0.751**	**0.986**
**Neves et al. 2016**	***DNMT1 rs16999593***	**TT vs TC+CC**	**1.31 (1.08–1.60)**	**0.00**	**0.908**	**0.899**	**0.995**
**Li et al. 2017**	***DNMT1 rs16999593***	**TC/CC vs. TT**	**1.36 (1.15–1.60)**	**0.00**	**0.881**	**0.191**	**0.891**
		**Cvs. T**	**1.28 (1.11–1.47)**	**0.00**	**0.988**	**0.323**	**0.951**
**Wang et al. 2019**	***DNMT3B rs1569686***	**T vs G**	**1.69 (1.36–2.10)**	**0.00**	**0.141**	**0.015**	**0.096**
		**TT vs TG**	**1.76 (1.38–2.24)**	**0.00**	**0.097**	**0.043**	**0.165**
		**TT vs TG+GG**	**1.78 (1.41–2.25)**	**0.00**	**0.076**	**0.018**	**0.066**

The DNMT1 rs16999593 variant, located at position 65 in exon 4, results in the substitution of a histidine for an arginine at position 97 in the amino acid sequence, which leads to a decrease in the expression of the DNMT1 gene. Another variant, DNMT3A rs1550117, located 448 base pairs upstream of the transcription start site, significantly reduces the transcriptional activity of the DNMT3A gene, resulting in a down-regulation of DNMT3A expression. On the other hand, the functional role of the DNMT3B rs1569686 variant, which is located at -579 base pairs from the transcription start site in exon 1B, remains controversial. It may affect the binding activity of multiple transcription factors. In this meta-analysis, we have incorporated 15 studies, out of which five delved into the connection between rs16999593 and the risk of gastric cancer, four studies investigated the rs1550117 polymorphism, and seven studies were related to the rs1569686 polymorphism. We also compared five genetic models. Our analysis revealed that DNMT1 rs16999593 is not significantly associated with the risk of gastric cancer. However, given the limited number of studies and the lack of studies from populations outside China, further research in diverse populations is required to validate the correlation between rs16999593 and gastric cancer. With regards to DNMT3A rs1550117, we discovered that AAvs. AG+GG was linked to an augmented risk of gastric cancer. When individual studies were excluded for heterogeneity analysis, we found that the study by Fan et al [32] was the source of heterogeneity. In the case of DNMT3A rs1569686, multiple gene models were connected to a decreased risk of gastric cancer. When we performed the heterogeneity analysis, we observed a significant decrease in overall heterogeneity when the study by Wang et al [21] was excluded. This meta-analysis employed multiple subgroups and various genetic models, leading to multiple comparisons. Therefore, the pooled p-values had to be adjusted [44]. The Venice criteria, statistical efficacy, and *I*^*2*^ values are crucial criteria [30]. Thus, we evaluated positive outcomes using the FPRP test and the Venice criterion. After conducting a confidence assessment, we identified statistically significant correlations with "low confidence in positive results" in the current meta-analysis.

This Meta-analysis study has the following strengths: (i) we comprehensively searched all major repositories and manually screened articles to minimize the omission of any studies relevant to the topic, regardless of language or study year; (ii) for included studies, we performed quality assessment; (iii) for controls, we performed the HWE test; (iv) we used the FPRP and Venice criteria to assess significant associations in the current Meta-analysis; and (v) we used a larger sample size than in the previous Meta-analysis. However, this study still has some limitations. First, we did not control for confounding factors such as H. pylori infection, smoking, and alcohol consumption, which may have influenced the results. Second, we observed significant heterogeneity in some genetic models. Although we performed publication bias testing, sensitivity analyses, and subgroup analyses to clarify the sources, we were unable to find all potential factors. Third, the language of publications was limited to English and Chinese. Fourth, although this meta-analysis was based on the whole population, most of the studies were from the Chinese population, except for one Iranian case, pending results from other populations in future studies.Finally, the small number of studies per SNP made it difficult to draw strong conclusions, and further studies are needed to determine the generalizability of these findings.

## Conclusion

After conducting a thorough analysis of 15 articles examining SNPs in DNMTs, we have concluded that DNMT1 rs16999593 does not exhibit a significant association with GC. However, our findings suggest that DNMT3A rs1550117 may be positively associated with GC, while DNMT3B rs1569686 may serve as a protective factor against GC. These SNPs could serve as valuable biomarkers to predict the risk of GC development and aid in the development of timely prevention strategies. However, further studies in diverse populations are necessary to confirm the association between these SNPs and GC risk. Additionally, investigating the biological significance of functional SNPs in DNMT activity and expression is essential for a comprehensive understanding of the impact of these SNPs on GC.

## Supporting information

S1 Checklist Plosone-checklist. (PDF)

S2 Checklist PRISMA 2020 checklist. (DOC)

S1 Table Scale for quality assessment of molecular association studies of gastric cancer. (DOCX)

S2 Table Included studies of DNMTs polymorphisms in gastric cancer within the meta-analyses. (DOCX)

S3 Table Characteristics of 14 studies included in the meta-analysis. (DOCX)
