# Supplementary material for: An updated meta-analysis investigating the association between DNMTs gene polymorphism andgastric cancer risk
Source: PLoS One. 2023 Oct 25;18(10):e0293466. doi: 10.1371/journal.pone.0293466 (PMC10599511; doi:10.1371/journal.pone.0293466)
Supplement: S1 Checklist — (PDF) [file pone.0293466.s001.pdf]

Meta-analysis on Genetic Association Studies Checklist | PLOS ONE

	Item	Section name and paragraph number within manuscript
	**Introduction**	
1	Provide a detailed justification for the polymorphism studied; if a single polymorphism was analyzed, give details as to why others were not included in the meta-analysis.	Introduction<br>Page2,43-51
2	Provide a detailed justification for the population(s) and clinical condition studied.	Introduction<br>Page1,24-32
	**Methods**	
3	Provide full details of the search strategy employed; outline the full electronic search strategy –specific combination of keywords and any limits applied- for at least one database. Specify whether synonyms of polymorphisms/genes (e.g. SNP number) were searched.	Search strategy<br>Page2,54-60
4	Report full details on the inclusion and exclusion criteria applied for selecting studies. *Please list the excluded articles and the reasons for exclusion of each article in a supplementary file.*	Selection criteria<br>Page2,62-65
5	Provide details on how the quality of the studies included in the analyses was assessed.	Quality assessment<br>Page3,76-80
6	Describe steps taken to contact study authors to identify additional studies and to request missing data.	Data extraction<br>Page2,67-74
7	Describe how environmental effects were adjusted for, if this adjustment was not conducted, outline the reasons for this.	Statistical analysis<br>Page3,94-96
8	Describe the methods of handling heterogeneity/between-study variance.	Statistical analysis<br>Page3,87-90

9	Describe how the Hardy-Weinberg equilibrium and linkage disequilibrium were assessed.	Statistical analysis<br>Page3,92-93
10	Describe and justify the choice of model for the analyses (per-allele vs per-genotype vs genetic model-free, random effects vs fixed effects).	Statistical analysis<br>Page3,84-86
11	Describe whether a sensitivity analysis has been completed.	Statistical analysis<br>Page3,90-94
12	Describe whether an assessment of the effects of population stratification has been conducted.	-
13	Describe whether study-specific results have been assessed and if so the reasons for this (e.g. forest plot).	Statistical analysis<br>Page3,95-98
	**Results**	
14	Include flow diagram for the studies included in the meta-analysis as the first figure for the manuscript	Description of included studies<br>Page3,109
15	Report details on allele/genotype prevalence.	Meta-analysis results<br>Page4,112-122
16	Report the effect size estimates and p values for each analysis.	Meta-analysis results<br>Page4,112-122
	**Discussion**	
17	Discuss the limitations of the meta-analysis, including genotyping errors/bias and publication bias.	Discussion<br>Page6,206-214
18	If the meta-analysis identifies an association within a subgroup of the population studied but not another, discuss the implications of	-

	these results, and if applicable the possibility of subgroup-specific publication bias.	
19	Discuss the suitability of the sample size employed to the research question and the power of the study.	**Discussion**<br>Page5,189-201
