# Supplementary material for: An updated meta-analysis investigating the association between DNMTs gene polymorphism andgastric cancer risk
Source: PLoS One. 2023 Oct 25;18(10):e0293466. doi: 10.1371/journal.pone.0293466 (PMC10599511; doi:10.1371/journal.pone.0293466)
Supplement: S1 Table — (DOCX) [file pone.0293466.s003.docx]

**Supplemental Table1** Scale for quality assessment of molecular association studies of gastric cancer

| Criterion | Score |
| --- | --- |
| Source of case | |
| Selected from population or cancer registry | 2 |
| Selected from hospital | 1 |
| Not described | 0 |
| Source of control | |
| Population-based | 2 |
| Hospital-based | 1 |
| Not described | 0 |
| Ascertainment of cancer | |
| Histological or pathological confirmation | 2 |
| Diagnosis of gastric cancer by patient medical record | 1 |
| Not described | 0 |
| Ascertainment of control | |
| Controls were tested to screen out gastric cancer | 2 |
| Controls were subjects who did not report gastric cancer, no objective testing | 1 |
| Not described | 0 |
| Matching | |
| Controls matched with cases by age and sex | 2 |
| Controls matched with cases only by age or sex | 1 |
| Not matched or not described | 0 |
| Genotyping examination | |
| Genotyping done blindly and quality control | 2 |
| Only genotyping done blindly or quality control | 1 |
| Unblinded and without quality control | 0 |
| HWE | |
| HWE in the control group | 2 |
| Hardy-Weinberg disequilibrium in the control group | 0 |
| Association assessment | |
| Assess association between genotypes and gastric cancer with appropriate statistics and adjustment for confounders | 2 |
| Assess association between genotypes and gastric cancer with appropriate statistics without adjustment for confounders | 1 |
| Inappropriate statistics used | 0 |
| Total sample size |  |
| ≥200 | 2 |
| <200 | 0 |

HWE: Hardy-Weinberg equilibrium
