# Supplementary material for: An updated meta-analysis investigating the association between DNMTs gene polymorphism andgastric cancer risk
Source: PLoS One. 2023 Oct 25;18(10):e0293466. doi: 10.1371/journal.pone.0293466 (PMC10599511; doi:10.1371/journal.pone.0293466)
Supplement: S2 Table — (DOCX) [file pone.0293466.s004.docx]

| **First Author/Year** | **Country** | **Eligible research studies of DNMT1 （rs16999593）** | | | **Eligible research studies of DNMT3A(rs1550117)** | | | | | **Eligible research studies of DNMT3B(rs1569686)** | | | | |
| --- | --- | --- | --- | --- | --- | --- | --- | --- | --- | --- | --- | --- | --- | --- |
|  |  | **All studies** | | | **All studies** | | | | | **All studies** | | | | |
|  |  | **This study** | **Li et al. 2016** | **Neves et al. 2016** | **This study** | **Li et al. 2016** | **Neves et al. 2016** | **Li et al. 2017** | **Wang et al. 2019** | **This study** | **Li et al. 2016** | **Neves et al. 2016** | **Chen et al. 2017** | **Wang et al. 2019** |
| Zhang et al. 2008 | Jiangsu/(China) |  | – | – |  | – | – | – |  | A | A | – | – | – |
| Fan et al. 2010 | Jiangsu/(China) |  | – | – | A | A | A | – | A |  | – | – | – | – |
| Hu et al. 2010 | Jiangsu/(China) |  | – | – |  | – | – | – |  | A | A | A | A | A |
| Yang et al., 2012 | Jiangxi(China) | A | A | A | A | A | A | – | A |  | – | – | – | – |
| Jiang et al.2012 | Jilin(China) | A | A | A |  | – | – | – |  |  | – | – | – | – |
| Cao et al. 2013 | Jilin(China) |  | – | – | A | A | A | – | A |  | – | – | – | – |
| Zhang et al.2014 | Heilongjiang(China) |  | – | – |  | – | – | – |  | A | A | – | A | A |
| Wang et al.2015 | Jilin(China) |  | – | – |  | – | – | – |  | A | A | A | – | – |
| Gao et al.2015 | Shandong(China) | A |  | – |  | – | – | – |  |  | – | – | – | – |
| Chen et al. 2017 | Hubei(China) |  | – | – |  | – | – | – |  |  | – | – | A | – |
| Ahmadi et al. 2017 | Lorestan（Iran） |  | – | – |  | – | – | – |  | A | – | – | – | – |
| Zhou et al. 2018 | Jiangsu/(China) | A | – | – | A | – | – | – |  |  | – | – | – | – |
| Liu et al. 2018 | Inner Mongolia(China) | A | – | – |  | – | – | – |  |  | – | – | – | – |
| Wang et al. 2019 | Hubei(China) |  |  |  |  | – | – | – |  | A | – | – | – | A |

**Supplemental Table 2** Included studies of DNMTs polymorphisms in gastric cancer within the meta-analyses
