# Supplementary material for: An updated meta-analysis investigating the association between DNMTs gene polymorphism andgastric cancer risk
Source: PLoS One. 2023 Oct 25;18(10):e0293466. doi: 10.1371/journal.pone.0293466 (PMC10599511; doi:10.1371/journal.pone.0293466)
Supplement: S3 Table — (DOCX) [file pone.0293466.s005.docx]

**Supplemental Table 3** Characteristics of 14 studies included in the meta-analysis. meta-analysis.

| **First author/Year** | **Province/Country** | **Area** | **Source of**  **controls** | **Type of control** | **Sample size** | **Genotypes distribution of DNMT1（rs16999593）** | | | | | | **HWE** | **Quality score** | **Genotypes distribution of DNMT3A(rs1550117)** | | | | | | **HWE** | **Quality score** | **Genotypes distribution of DNMT3B(rs1569686)** | | | | | | **HWE** | **Quality score** |
| --- | --- | --- | --- | --- | --- | --- | --- | --- | --- | --- | --- | --- | --- | --- | --- | --- | --- | --- | --- | --- | --- | --- | --- | --- | --- | --- | --- | --- | --- |
|  |  |  |  |  |  | **Cases** | | | **Controls** | | |  |  | **Cases** | | | **Controls** | | |  |  | **Cases** | | | **Controls** | | |  |  |
|  |  |  |  |  |  | **CC** | **CT** | **TT** | **CC** | **CT** | **TT** |  |  | **GG** | **AG** | **AA** | **GG** | **AG** | **AA** |  |  | **GG** | **GT** | **TT** | **GG** | **GT** | **TT** |  |  |
| Zhang et al. 2008[31] | Jiangsu/(China) | South China | HB | Non-gastric cancer Controls | 156/156 | – | – | – | – | – | – | – | – | – | – | – | – | – | – | – | – | 2 | 18 | 136 | 7 | 30 | 119 | 0.0100 | 9（HWD） |
| Fan et al. 2010[32] | Jiangsu/(China) | South China | HB | Healthy controls | 208/364 | – | – | – | – | – | – | – | – | 102 | 75 | 31 | 218 | 118 | 10 | 0.2055 | 13 | – | – | – | – | – | – | – | – |
| Hu et al. 2010[33] | Jiangsu/(China) | South China | HB | Healthy controls | 259/262 | – | – | – | – | – | – | – | – | – | – | – | – | – | – | – | – | 2 | 27 | 230 | 4 | 55 | 203 | 0.9011 | 13 |
| Yang et al., 2012[13] | Jiangxi(China) | South China | HB | Non-gastric cancer Controls | 242/294 | 12 | 89 | 141 | 15 | 83 | 196 | 0.1197 | 12 | 157 | 74 | 11 | 191 | 93 | 10 | 0.7472 | 12 | – | – | – | – | – | – | – | – |
| Jiang et al.2012[34] | Jilin(China) | North China | PB | Healthy controls | 447/961 | 20 | 144 | 283 | 29 | 273 | 659 | 0.7580 | 14 | – | – | – | – | – | – | – | – | – | – | – | – | – | – | – | – |
| Cao et al.2013[35] | Jilin(China) | North China | PB | Healthy controls | 447/961 | – | – | – | – | – | – | – | – | 289 | 142 | 16 | 640 | 288 | 33 | 0.9316 | 14 | – | – | – | – | – | – | – | – |
| Zhang et al.2014[36] | Heilongjiang(China) | North China | HB | Healthy controls | 50/60 | – | – | – | – | – | – | – | – | – | – | – | – | – | – | – | – | 0 | 7 | 43 | 0 | 12 | 48 | 0.3894 | 6 |
| Wang et al.2015[37] | Jilin(China) | North China | PB | Healthy controls | 447/961 | – | – | – | – | – | – | – | – | – | – | – | – | – | – | – | – | 5 | 82 | 360 | 10 | 150 | 801 | 0.3207 | 9 |
| Gao et al.2015[14] | Shandong(China) | North China | PB | Healthy controls | 310/420 | 18 | 112 | 180 | 22 | 117 | 281 | 0.0385 | 9（HWD） | – | – | – | – | – | – | – | – | – | – | – | – | – | – | – | – |
| Chen et al.2017[20] | Hubei(China) | South China | PB | Non-gastric cancer Controls | 460/800 | – | – | – | – | – | – | – | – | – | – | – | – | – | – | – | – | 5 | 81 | 374 | 16 | 204 | 580 | 0.6928 | 14 |
| Ahmadi et al.2017[38] | Lorestan（Iran） | Iran | HB | Healthy controls | 100/112 | – | – | – | – | – | – | – | – | – | – | – | – | – | – | – | – | 45 | 27 | 28 | 46 | 44 | 22 | 0.0618 | 12 |
| Zhou et al.2018[17] | Jiangsu/(China) | South China | HB | Healthy controls | 466/452 | 18 | 149 | 299 | 21 | 139 | 292 | 0.3976 | 12 | 299 | 150 | 17 | 287 | 147 | 18 | 0.8790 | 12 | – | – | – | – | – | – | – | – |
| Liu et al.2018[22] | Inner Mongolia(China) | North China | PB | Healthy controls | 381/427 | 15 | 87 | 277 | 14 | 125 | 284 | 0.9568 | 13 | – | – | – | – | – | – | – | – | – | – | – | – | – | – | – | – |
| Wang et al.2019[21] | Hubei(China) | South China | HB | Healthy controls | 460/1798 | – | – | – | – | – | – | – | – | – | – | – | – | – | – | – | – | 5 | 81 | 374 | 16 | 204 | 580 | 0.6928 | 10 |

**HB = hospital-based studies, PB = population-based studies，HWE= Hardy-Weinberg equilibrium.**
